# Ultrasonographic Evaluation of Hypervolemic and Normovolemic Patients: A Comparison of Inferior Vena Cava, Subclavian Vein, Internal Jugular Vein, and Femoral Vein Diameters and Collapsibility Indices

**DOI:** 10.7759/cureus.77488

**Published:** 2025-01-15

**Authors:** İsmail Ataş, Mümin Murat Yazıcı, Enes Hamdioğlu, Nurullah Parça, Meryem Kaçan, Özcan Yavaşi, Özlem Bilir

**Affiliations:** 1 Department of Emergency Medicine, Recep Tayyip Erdoğan University Training and Research Hospital, Rize, TUR

**Keywords:** femoral vein, internal jugular vein, normovolemia, pocus, subclavian vein, volume overload

## Abstract

Background and objective

We aimed to determine the diameters and respiratory variability of the subclavian vein (SCV), internal jugular vein (IJV), and femoral vein (FV), which are more superficial and easier to visualize with point-of-care ultrasound (PoCUS) in the detection of volume overload, and to investigate whether they can be an alternative to analyzing an inferior vena cava (IVC) to determine volume load.

Methodology

We prospectively evaluated volume-overloaded and normovolemic patients admitted to the emergency department using PoCUS for six months. Inspiratory-expiratory diameters and collapsibility indices (CI) of IVC and SCV, IJV, and FV were evaluated. The correlation between IVC and SCV, IJV, and FV was analyzed.

Results

A total of 176 patients were included in the study, including 88 volume-overloaded patients in the study group and 88 normovolemic patients in the control group. The median values of the maximum and minimum diameters of the IVC, SCV, IJV, and FV in the study group were statistically higher compared to the control group. A moderate correlation was found between IVC and SCV, IVC and IJV, and IVC and FV for maximum diameters in all patients (p = 0.447, p = 0.515, and p = 450, respectively). There was a very weak correlation between the IVC-CI and the FV-CI in all patients (p = 0.160), and no correlation was found with the other veins.

Conclusion

The IVC-CI was not correlated with the SCV-CI, the IJV-CI, or the FV-CI in volume-overloaded patients; therefore, superficial venous vessels cannot be an alternative to the IVC.

## Introduction

The volume status of critically ill patients admitted to emergency departments (EDs) should be determined quickly and effectively. Various clinical signs and symptoms are used to determine this amount of patients. Volume overload is estimated by physical examination findings, such as peripheral edema, the presence of pulmonary rales, and jugular venous filling [1-3]. These are subjective and vary according to a clinician’s assessment. A hypervolemia status cannot be determined with certainty; therefore, various methods are needed to determine this [4].

The gold standard method for determining a person’s volume status is using a Swan-Ganz catheter to directly measure capillary wedge pressure [5,6]. This method is invasive and difficult to apply in hemodynamically unstable patients. In recent years, point-of-care ultrasound (PoCUS), which is a more practical, easily applicable, inexpensive, and noninvasive method, has been used [7]. In addition, ultrasound is a good noninvasive alternative to the Swan-Ganz catheter to assess cardiac function and determine the volume status [8]. The diameter of the inferior vena cava (IVC), a major vein that best reflects cardiac preload, and a diameter change that occurs in this venous structure with respiration are used to determine hypervolemia status with PoCUS [9-11].

Sometimes, an IVC may not be evaluated properly in obese patients with volume overload or in the abdomen due to dense intraintestinal gas [12]. The use of other superficial central venous structures that can be visualized more easily with PoCUS as an alternative to the IVC should be investigated. In this study, we aimed to determine the diameters and respiratory variability (collapsibility index (CI)) of the subclavian vein (SCV), internal jugular vein (IJV), and femoral vein (FV), which are more superficial and easier to visualize with PoCUS in the detection of hypervolemia, and to investigate whether they can be an alternative to analyzing an IVC to determine volume overload.

## Materials and methods

Study design and setting

This prospective, cross-sectional, single-center study was conducted in a tertiary care training and research hospital in Turkey between June 1, 2024, and November 30, 2024.

Patient selection and data collection

The patient group was formed by including all patients with volume overload admitted to the ED’s critical care area and excluding patients who met the exclusion criteria for this study. Between June 1, 2024, and September 30, 2024, patients with volume overload admitted to the ED’s critical care area were included. The following patients were excluded from this study: one patient under 18 years of age; 12 patients who did not give consent for this study and withdrew; eight patients who could not undergo an ultrasound (US) for any reason (i.e., equipment unavailable, staff not available, etc.); 28 patients in whom the measurements of venous structures may have affected in the US (i.e., invasive and noninvasive mechanical ventilation, presence of thrombus in venous structures); nine patients admitted to the ED due to cardiac arrest due to potentially hypovolemic conditions; 19 patients admitted because of trauma due to potentially hypovolemic conditions; and six patients without appropriate measurements detected by a specialist with PoCUS experience on recorded US images.

**Figure 1:**
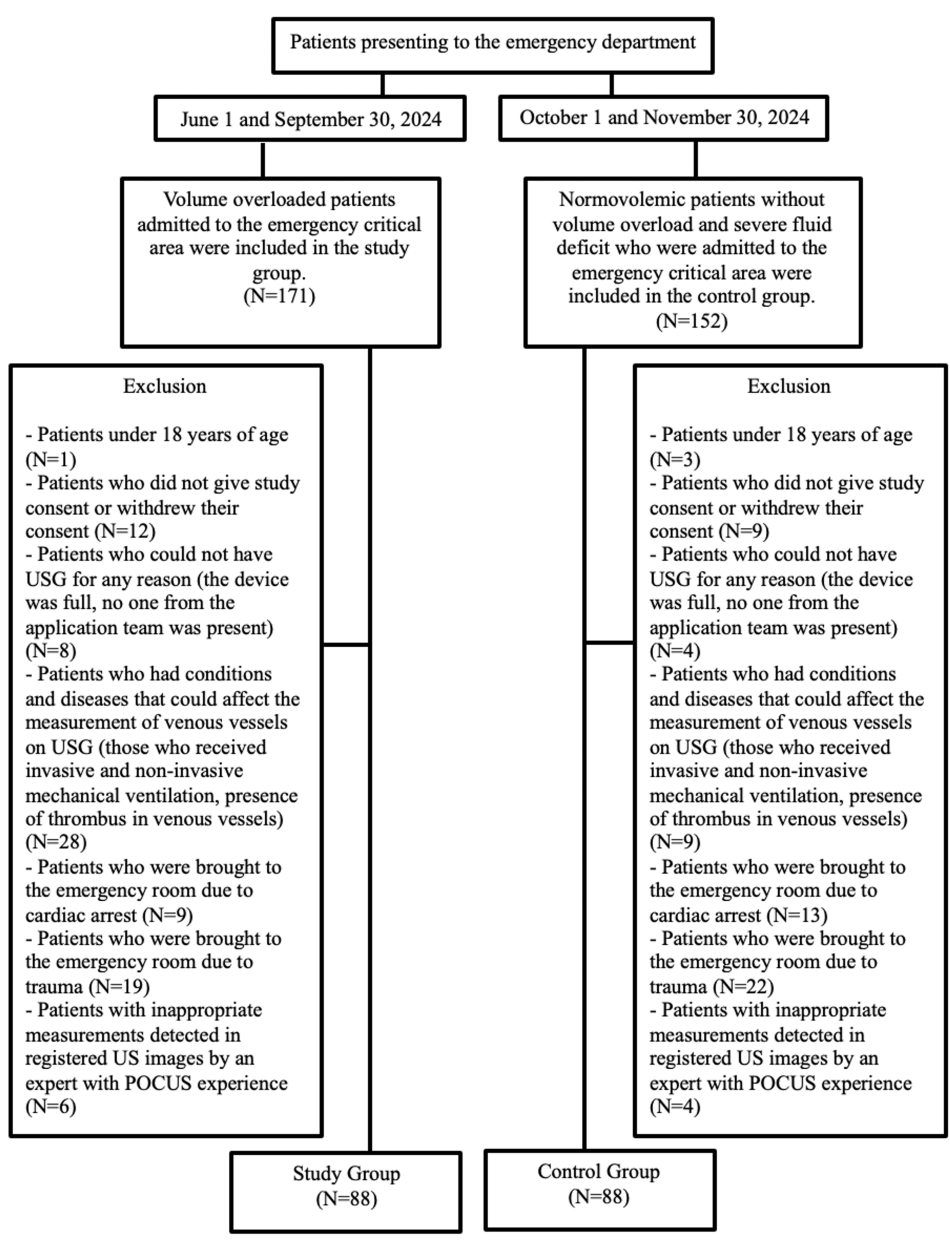
Flowchart of the patients. US: ultrasound; USG: ultrasonography; POCUS: point-of-care ultrasound.

After applying the exclusion criteria, 83 patients admitted to the critical area of the ED were excluded, and 88 patients were identified as the patient group. Also, as a control group, between October 1, 2024, and November 30, 2024, the first 88 normovolemic patients without volume overload and severe fluid deficit were included. Those who met the exclusion criteria were removed, and a control group was formed. The following patients from the control group were excluded from this study: three patients under 18 years of age; nine patients who did not give consent for this study and withdrew; four patients who could not undergo a US for any reason (i.e., the equipment unavailable, staff not available, etc.); nine patients in whom the measurements of venous structures may have affected in the US; 13 patients admitted to the ED due to cardiac arrest due to potentially hypovolemic conditions; 22 patients admitted to the ED due to trauma due to potentially hypovolemic conditions; and four patients without appropriate measurements detected by a specialist with PoCUS experience on recorded US images. After applying the exclusion criteria, 64 patients admitted to the critical area of the ED were excluded from the study. The patient flowchart is shown in Figure 1.

Study protocol

Initial patient evaluations in the ED were performed by two emergency medicine residents with three years of training. The patient population of this study was defined as those who were considered to be volume overload (presence of nocturnal cough, orthopnea, and paroxysmal nocturnal dyspnea in the anamnesis; wheezing or rales in the lungs, pitting pretibial edema, jugular venous distension, and presence of a third heart sound on physical examination; increased cardiothoracic ratio, cardiomegaly, and presence of cephalization on X-ray; Kerley B lines, pleural effusion, and presence of ascites on bedside US) and were followed up with the prediagnoses of decompensated heart failure, pulmonary edema, chronic renal disease on dialysis, or planned to start diuretic treatment. Patients considered as the control group were normovolemic patients without volume overload and severe fluid deficits (dehydrated patients with dry mucosa, increased skin turgor, and no urine output). Patients with any mechanical ventilation device (invasive or noninvasive) that would affect the US measurement of venous vessels and patients with thrombus in venous structures were excluded from these selected patients. Other exclusion criteria were then applied. Finally, we excluded patients whose US measurements were inappropriate and who refused diagnosis and treatment.

The emergency medicine residents who performed the initial assessment recorded all vital and physical examination findings. The residents also recorded the demographic characteristics and comorbidities of the patients included in this study. Primary diagnoses and treatments were made and administered by the same emergency medicine residents who performed the initial evaluation.

US measurements of the patients’ venous vessels were performed by another two emergency medicine residents. The two residents who performed the clinical evaluation of the patients and the two residents who performed the US measurements were blinded to each other. Both had attended and completed a US course (basic) approved by professional emergency medicine associations (three years of experience in PoCUS, with an average of 250 USs per year). Two emergency medicine specialists who had no responsibility for the primary care of the patients analyzed the data. One emergency medicine specialist checked the vital signs, physical examinations, diagnosis, and treatments, while the other emergency medicine specialist checked the US measurements. Emergency specialists were blinded among themselves and with emergency medicine residents.

Ultrasound protocol

Sonographic examinations were performed on a FUJIFILM SonoSite FC1 (FUJIFILM SonoSite, Inc., Bothell, WA) 2015 model US device. IVC measurements were performed with a 1-5 megahertz (MHz) sector (cardiac) probe. The patient was placed in the supine position, and the IVC was imaged in grayscale mode (B-mode) and motion mode (M-mode) using a transabdominal long-axis window from the subxiphoid region. For measurement, the IVC was measured 2 cm caudal from the entry point of the heart into the right atrium, and the widest and narrowest diameters were determined as the inspiratory and expiratory diameters using the M-mode. These measurements were repeated three times and averaged. The respiratory variability of the IVC was determined as a percentage and defined as the collapsibility index of the inferior vena cava (IVC-CI), and it was calculated as follows: [(Maximum IVC diameter - Minimum IVC diameter)/Maximum IVC diameter] × 100.

SCV, IJV, and FV measurements were performed with a 9-12 MHz linear probe. To evaluate an SCV, the patient was placed in the supine position. The probe was placed perpendicular to the patient’s body on the upper edge of the right clavicle, parallel to the long axis of the clavicle, with the notch facing the patient’s head. A longitudinal image of the SCV was obtained. The patient was placed in the supine position to evaluate the IJV. The patient’s head was turned in the opposite direction to the side to be evaluated. The probe was placed on the anterior line of the sternocleidomastoid (SCM) muscle parallel to the SCM muscle and oblique to the long axis of the neck, with the notch facing the cranial side. Thus, a longitudinal view of the IJV was obtained. To evaluate the FV, the probe is placed just below the inguinal ligament, parallel to the ligament, oblique to the body, with the notch facing cranially. When the short axis of the femoral artery-vein was seen, the probe was turned 90°, and the longitudinal axis of the FV was obtained.

For SCV, IJV, and FV, the widest and narrowest diameters were determined as inspiratory and expiratory diameters with M-mode, and measurements were made. These measurements were repeated three times and averaged. The respiratory variability of all veins was determined as a percentage, and the CI was calculated. The recording procedure started when the US windows provided by the images were clearly visible and ended when the desired measurements were completed.

Study endpoint

The primary aim of this study was to evaluate the US correlation of the inspiratory-expiratory diameters and collapsibility indices of the IVC and other major superficial venous vessels (SCV, IJV, and FV) in hypervolemic and normovolemic patients.

Statistical analysis

All analyses were performed using Jamovi version 1.6 statistical software (The Jamovi Project, Sydney, Australia). Categorical data are expressed as frequencies (n) and percentages. Normally distributed continuous variables are presented as a mean plus standard deviation, and non-normally distributed data are presented as a median and interquartile range (IQR). The normality of distribution was assessed using the Shapiro-Wilk test. Continuous variables were compared in independent groups using a t-test in the case of normal distribution and a Mann-Whitney U test in the case of non-normal distribution. Comparisons between categorical data were evaluated using a chi-squared test. Pearson correlation for normally distributed variables and Spearman correlation analysis for non-normally distributed variables were performed to evaluate the relationships between the US measurements. In all statistical analyses, p-values <0.05 were considered significant.

Ethical statement

Ethical approval for this study was provided by the Recep Tayyip Erdoğan University Ethics Committee (Approval No.: E-40465587-050.01.04-815; ID: 2023/214; dated: September 20, 2023). All the patients we planned to include in this study were told how and for what purpose a bedside ultrasonography would be performed, and written informed consent was obtained from all patients who agreed to participate in this study. To protect the identities of the patients, all data collected were made anonymous, and patients were informed of their right to participate without coercion and that they could even cease participating without regret in their medical treatment.

## Results

Of the patients included in this study, 55.1% (N = 97) were male. The median age of the patients was 78.5 years (IQR = 69.8-84) in the study group and 67 years (IQR = 55.8-78.3) in the control group.

When the patients’ comorbidities were evaluated, hypertension (41.5%), diabetes (15.3%), chronic heart failure (35.8%), atrial fibrillation (22.3%), and chronic renal failure (9.1%) were higher in patients with volume overload than in normovolemic patients. When the vital signs of all patients were evaluated, systolic blood pressure, diastolic blood pressure, and pulse rate were higher in the study group, and oxygen saturation was lower in the study group compared to the control group. All the patients in the study group had pretibial pitting edema and pulmonary rales, whereas none of the patients in the control group had these findings. The demographic data of the patients are shown in Table 1.

**Table 1:** Demographic data and baseline characteristics of the study and control groups. *: Chi-squared test; ^: Mann‒Whitney U test; IQR: interquartile range (25p, 75p); sd: standard deviation; CAD: coronary artery disease; COPD: chronic obstructive pulmonary disease; CHF: congestive heart failure; CRF: chronic renal failure; SBP: systolic blood pressure; DBP: diastolic blood pressure; SpO2: oxygen saturation.

Characteristics	All patients (N = 176)	Study group (N = 88)	Control group (N = 88)	p-value
Gender				
Male, n (%)	97 (55.1%)	44 (25.0%)	53 (30.1%)	0.173*
Female, n (%)	79 (44.9%)	44 (25.0%)	35 (19.9%)	
Age (years), median (IQR)	73.5 (63-83)	78.5 (69.8-84)	67 (55.8-78.3)	0.001^
Comorbidities				
Hypertension, n (%)	111 (63.1%)	73 (41.5%)	38 (21.6%)	0.001*
Diabetes, n (%)	42 (23.9%)	27 (15.3%)	15 (8.6%)	0.034*
CAD, n (%)	48 (27.3%)	29 (16.5%)	19 (10.8%)	0.091*
COPD, n (%)	15 (8.6%)	11 (6.3%)	4 (2.3%)	0.059*
CHF, n (%)	66 (37.5%)	63 (35.8%)	3 (1.7%)	0.001*
Atrial fibrillation, n (%)	50 (28.6%)	39 (22.3%)	11 (6.3%)	0.001*
CRF, n (%)	20 (11.4%)	16 (9.1%)	4 (2.3%)	0.004*
Vital signs				
SBP (mmHg), median (IQR)	139 (120-160)	142 (126-170)	132 (120-144)	0.001^
DBP (mmHg), median (IQR)	80 (70-90)	80 (70-98.3)	77 (70-81)	0.009^
Pulse (/min), median (IQR)	85 (75-100	89.5 (79-105)	79.5 (70.8-95.3)	0.001^
SpO_2 _(%), median (IQR)	95 (90-98)	92 (86-95)	97 (95-98)	0.001^
Examination findings				
Pitting edema, n (%)	88 (50.0%)	87 (49.4%)	1 (0.6%)	0.001*
Rales, n (%)	88 (50.0%)	87 (49.4%)	1 (0.6%)	0.001*
Urine output, n (%)	174 (98.9%)	88 (50.0%)	86 (48.9%)	0.155*

When the US measurements of the patients were evaluated, the median values of the maximum and minimum diameters of the IVC in the study group were 2.53 and 1.89 cm, respectively. These values were statistically higher compared to the control group (p = 0.001 and p = 0.001, respectively). Similarly, the median values of the maximum and minimum diameters of the SCV, IJV, and FV in the study group were also significantly higher than those in the control group. Although the median values of the IVC-CI, SCV-CI, and FV-CI were lower in the study group, statistical significance was not found (p = 0.658, p = 0.728, and p = 306, respectively). The IJV-CI was higher in the study group, but again, no statistical significance was found (p = 0.146) (Table 2).

**Table 2:** Ultrasound measurements of the study and control groups. ^: Mann‒Whitney U test; IQR: interquartile range (25p, 75p); CI: collapsibility index; IVC: inferior vena cava; SCV: subclavian vein; IJV: internal jugular vein; FV: femoral vein.

Ultrasound measurements	All patients (N = 176)	Study group (N = 88)	Control group (N = 88)	p-value
IVC maximum (cm), median (IQR)	2.12 (1.76-2.58)	2.53 (2.21-2.80)	1.78 (1.5-2.03)	0.001^
IVC minimum (cm), median (IQR)	1.62 (1.23-194)	1.89 (1.59-2.15)	1.31 (0.98-1.63)	0.001^
IVC-CI (%), median (IQR)	25 (16-32)	24.5 (16.8-33.0)	25 (16-30)	0.658^
SCV maximum (cm), median (IQR)	0.79 (0.63-0.99)	0.92 (0.76-1.04)	0.70 (0.56-0.82)	0.001^
SCV minimum (cm), median (IQR)	0.6 (0.47-0.82)	0.73 (0.55-0.89)	0.50 (0.42-0.66)	0.001^
SCV-CI (%), median (IQR)	18.5 (9.8-28)	17 (9-29)	20 (10-26.3)	0.728^
IJV maximum (cm), median (IQR)	0.86 (0.64-1.13)	1.04 (0.81-1.26)	0.67 (0.54-0.95)	0.001^
IJV minimum (cm), median (IQR)	0.65 (0.5-0.9)	0.79 (0.59-1.06)	0.54 (0.43-0.75)	0.001^
IJV-CI (%), median (IQR)	19.5 (13-28)	20 (13-31)	18 (13-24)	0.146^
FV maximum (cm), median (IQR)	1.13 (0.89-1.33)	1.25 (1.09-1.42)	1.0 (0.75-1.24)	0.001^
FV minimum (cm), median (IQR)	1.01 (0.79-1.26)	1.17 (0.90-1.36)	0.89 (0.64-1.17)	0.001^
FV-CI (%), median (IQR)	8 (4-17)	7 (4-17)	10 (4-16)	0.306^

**Figure 2:**
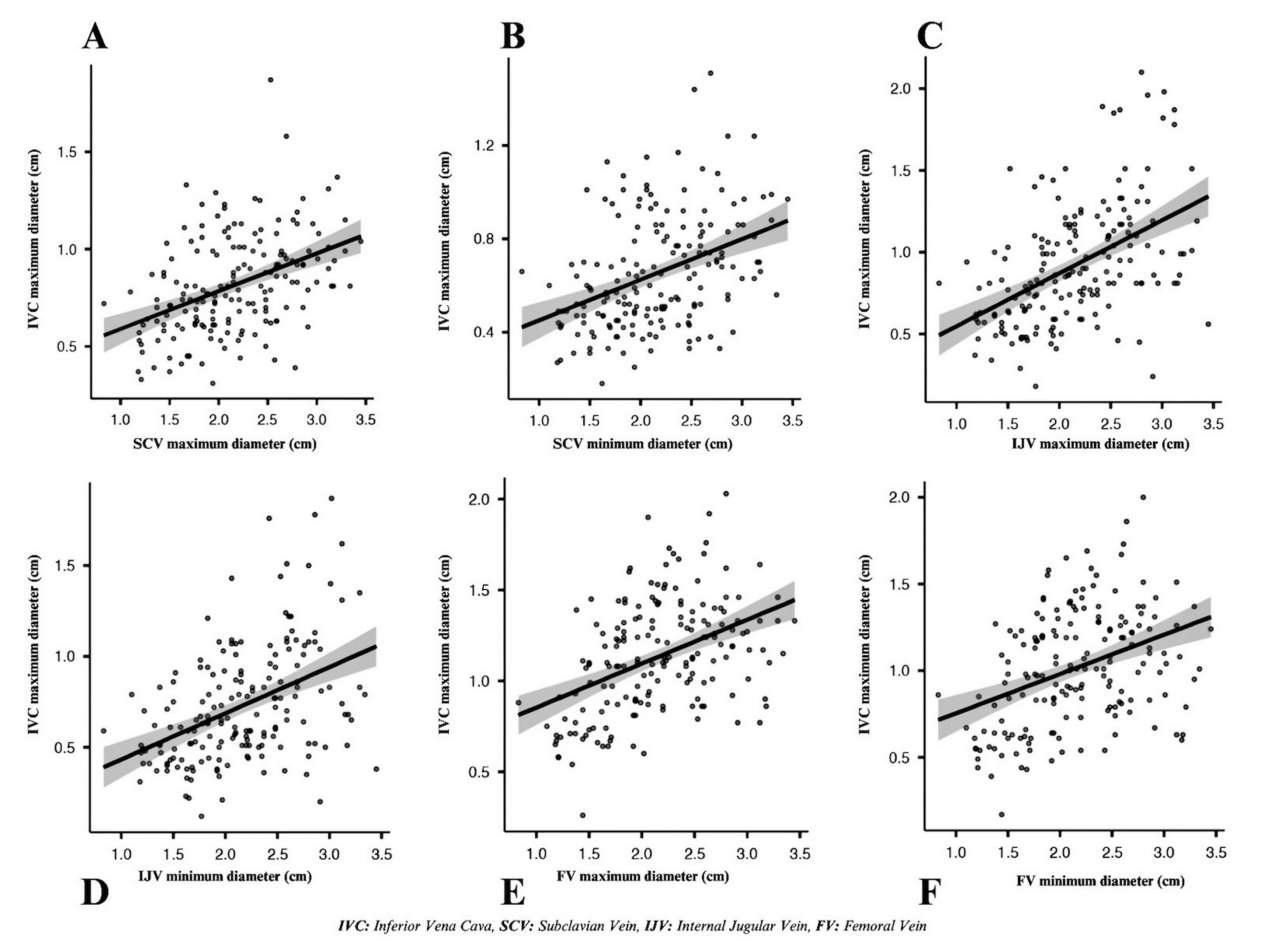
The correlation between IVC maximum diameters and maximum–minimum diameters of other venous vessels in all patients. (A) IVC maximum diameter-SCV maximum diameter (0.447). (B) IVC maximum diameter-SCV minimum diameter (0.416). (C) IVC maximum diameter-IJV maximum diameter (0.515). (D) IVC maximum diameter-IJV minimum diameter (0.445). (E) IVC maximum diameter-FV maximum diameter (0.450). (F) IVC maximum diameter-FV minimum diameter (0.387). IVC: inferior vena cava; SCV: subclavian vein; IJV: internal jugular vein; FV: femoral vein.

**Figure 3:**
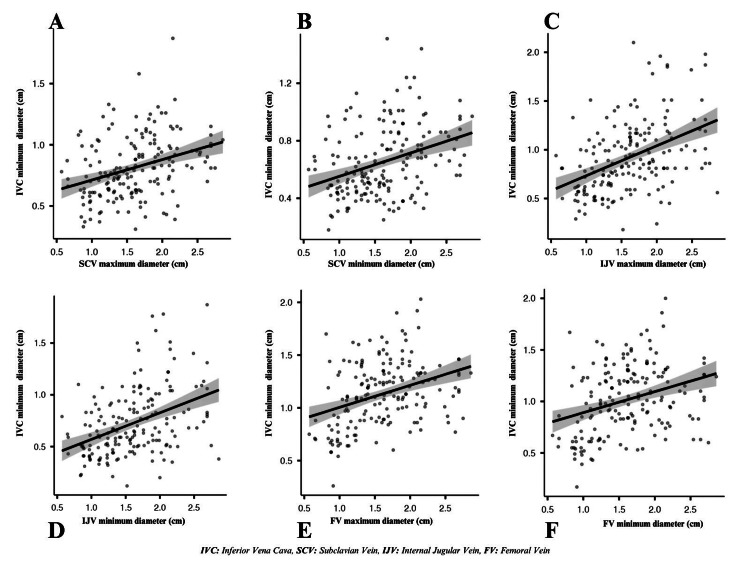
The correlation between IVC minimum diameters and maximum–minimum diameters of other venous vessels in all patients. (A) IVC minimum diameter-SCV maximum diameter (0.380). (B) IVC minimum diameter-SCV minimum diameter (0.363). (C) IVC minimum diameter-IJV maximum diameter (0.451). (D) IVC minimum diameter-IJV minimum diameter (0.419). (E) IVC minimum diameter-FV maximum diameter (0.371). (F) IVC minimum diameter-FV minimum diameter (0.334). IVC: inferior vena cava; SCV: subclavian vein; IJV: internal jugular vein; FV: femoral vein.

The correlation between IVC maximum diameters, minimum diameters, CI values, and the diameters and CI values of other central venous vessels (SCV, IJV, FV) in all patients, study group, and control group patients were analyzed separately (Table 3). A moderate correlation was found between IVC and SCV, IJV, and FV for maximum diameters in all patients (p = 0.447, p = 0.515, and p = 450, respectively). For minimum diameters, there was a weak correlation between the IVC, SCV, and FV, and a moderate correlation with the IJV in all patients (p = 0.363, p = 0.334, and p = 0.419, respectively). There was a very weak correlation between the IVC-CI and the FV-CI in all patients (p = 0.160); no correlation was found with the other veins. The correlation between IVC maximum diameters and maximum-minimum diameters of other venous vessels and the correlation between IVC minimum diameters and maximum-minimum diameters of other venous vessels in all patients are shown in Figures 2, 3.

**Table 3:** Correlation between ultrasound measurements. *: Spearman’s correlation analysis; CI: collapsibility index; IVC: inferior vena cava; SCV: subclavian vein; IJV: internal jugular vein; FV: femoral vein.

Total ultrasound measurements	Correlation coefficient (p-value*)		
	IVC maximum (cm)	IVC minimum (cm)	IVC-CI (%)
SCV maximum (cm)	0.447 (0.001)	0.380 (0.001)	-0.045 (0.550)
SCV minimum (cm)	0.416 (0.001)	0.363 (0.001)	-0.061 (0.422)
SCV-CI (%)	-0.115 (0.130)	-0.157 (0.037)	0.120 (0.114)
IJV maximum (cm)	0.515 (0.001)	0.451 (0.001)	-0.077 (0.312)
IJV minimum (cm)	0.445 (0.001)	0.419 (0.001)	-0.120 (0.112)
IJV-CI (%)	0.040 (0.594)	-0.004 (0.953)	0.082 (0.277)
FV maximum (cm)	0.450 (0.001)	0.371 (0.001)	-0.024 (0.754)
FV minimum (cm)	0.387 (0.001)	0.334 (0.001)	-0.067 (0.374)
FV-CI (%)	-0.172 (0.023)	-0.182 (0.015)	0.160 (0.034)
Study group ultrasound measurements			
SCV maximum (cm)	0.311 (0.003)	0.319 (0.002)	-0.177 (0.098)
SCV minimum (cm)	0.309 (0.003)	0.363 (0.001)	-0.261 (0.014)
SCV-CI (%)	-0.209 (0.051)	-0.258 (0.015)	0.193 (0.072)
IJV maximum (cm)	0.355 (0.001)	0.284 (0.007)	-0.063 (0.562)
IJV minimum (cm)	0.302 (0.004)	0.296 (0.005)	-0.133 (0.217)
IJV-CI (%)	0.023 (0.835)	-0.077 (0.478)	0.155 (0.148)
FV maximum (cm)	0.018 (0.868)	-0.074 (0.491)	0.132 (0.221)
FV minimum (cm)	-0.007 (0.951)	-0.083 (0.442)	0.096 (0.372)
FV-CI (%)	-0.038 (0.723)	0.015 (0.886)	-0.037 (0.732)
Control group ultrasound measurements			
SCV maximum (cm)	0.187 (0.081)	0.057 (0.599)	0.079 (0.465)
SCV minimum (cm)	0.118 (0.274)	-0.021 (0.845)	0.125 (0.244)
SCV-CI (%)	-0.009 (0.933)	-0.021 (0.849)	0.034 (0.757)
IJV maximum (cm)	0.253 (0.017)	0.259 (0.015)	-0.157 (0.144)
IJV minimum (cm)	0.227 (0.034)	0.243 (0.022)	-0.165 (0.124)
IJV-CI (%)	-0.111 (0.303)	-0.050 (0.642)	0.005 (0.960)
FV maximum (cm)	0.500 (0.001)	0.473 (0.001)	-0.233 (0.029)
FV minimum (cm)	0.468 (0.001)	0.466 (0.001)	-0.274 (0.010)
FV-CI (%)	-0.309 (0.003)	-0.475 (0.001)	0.381 (0.001)

In the study group, there was a weak correlation between the IVC, the SCV, and the IJV for maximum diameters (p = 0.311 and p = 0.355, respectively), and a weak correlation between the IVC and SCV, and IJV minimum diameters (p = 0.363 and p = 0.296, respectively). No correlation was found for the other measurements.

In the control group, there was a weak correlation between the IVC and the IJV maximum diameters (p = 0.253) and a moderate correlation between the IVC and the FV maximum diameters (p = 0.500). In the control group, there was a weak correlation between IVC and IJV for minimum diameters (p = 0.243) and a moderate correlation between IVC and FV (p = 0.466). In the control group, a weak correlation was found between the IVC-CI and the FV-CI (p = 0.381). No correlation was found for the other measurements.

## Discussion

Cardiac output and stroke volume should be known when determining the fluid volume of critically ill patients. The gold standard method used to determine this is the Swan-Ganz catheter, which is an invasive method that directly measures capillary wedge pressure [4]. This method is difficult and highly invasive, especially in unstable patients, and its use is limited in EDs where patients need to be diagnosed and intervened rapidly [13]. In recent years, PoCUS has been used to determine fluid volume. PoCUS is a noninvasive, practical, bedside method with a low cost [14]. It is a method that has become widespread in EDs in recent years and has been used to determine the diameter and the IVC-CI for determining a patient’s blood volume [15,16]. In this current study, we used PoCUS to investigate IVC, SCV, IJV, and FV diameters and CI in volume-overloaded and normovolemic patients.

In this current study, no correlation was found between the IVC-CI, SCV-CI, and IJV-CI in either the study group or the control group. A very weak correlation was found between the IVC-CI and the FV-CI in the control group. In a pilot study conducted on 36 patients with acute heart failure, a high correlation was found between the IVC-CI and the SCV-CI [17]. In another study, a correlation was found between IVC-CI and IJV-CI in 16 spontaneously breathing patients, but this correlation was said to disappear under positive pressure ventilation [18]. Our study does not overlap with the literature on this subject. In a study conducted on 102 patients with renal disease, a high correlation was found between the IVC-CI and the SCV-CI. In the same study, the IVC-CI was less than 20%, and the SCV-CI was less than 22% in patients with volume overload findings [19]. In this study, the IVC-CI and the SCV-CI in patients with volume overload were 24.5% and 17%, respectively. These values coincide with previous studies. When the literature was reviewed, no studies on FV were found, and further studies could be designed.

The study group in this current study consisted of volume-overloaded patients with a median IVC maximum diameter of 2.53 cm and a median CI of 24.5%. According to the guidelines published by the American Society of Echocardiography in 2010, patients can be considered volume overloaded if the maximum IVC diameter is greater than 2.1 cm and the CI is below 50% [20]. These values coincide with previous studies. In the control group, the median IVC maximum diameter was 1.78 cm; accordingly, we can say that they were not volume overloaded. Similarly, the maximum diameters of the SCV, IJV, and FV were statistically higher in the study group than in the control group. In a study conducted by Munir et al., SCV maximum diameters were measured in the supine position in 39 patients with acute heart failure, and a mean of 10.99 mm was found [21]. In this current study, the median SCV maximum diameter was 9.2 mm. When the literature was reviewed, no study was found regarding IJV and FV maximum diameters in patients with volume overload. We believe that multicentric and large studies on SCV, IJV, and FV maximum diameters in patients with volume overload are needed.

Many studies have shown that venous vessel diameters and respiratory variability are affected in patients on mechanical ventilation and give different results in determining the volume status of patients [22-25]. In some studies, the feasibility of measuring venous vessel diameters in mechanically ventilated patients has been mentioned [26,27]. In our study, patients receiving mechanical ventilation and patients with structural disorders (such as intravascular thrombus) that may affect venous vessel diameters were excluded because of the concern that this issue could affect our measurements due to a lack of clarity and to ensure standardization in measurements.

In our study, the median age of the study group was statistically significantly higher than that of the control group. At the same time, comorbid diseases, such as hypertension, diabetes, heart failure, atrial fibrillation, and chronic renal failure, were significantly higher in the study group than in the control group. In the literature, there are studies in which hypertension, diabetes, and chronic renal failure cause volume overload due to impaired renal function and atrial fibrillation, while heart failure causes volume overload with a cardiac effect [28-31]. Our study is correlated with the literature in this respect.

Kent et al. reported in a study that venous vessels close to the surface, such as the IJV and the SCV, may be exposed to external compression, whereas the SCV is protected by the clavicle due to its position and may be less exposed to this compression [32]. Since the IVC cannot be exposed to external compression, it has an advantage over the IJV, SCV, and FV. The disadvantage of the IVC compared to other venous vessels is that it is affected by intra-abdominal pressure.

Limitations

In this study, the ages of the study group were significantly higher than those of the control group. When forming the study population, the ages of the control group could be chosen close to the study group. In addition, the study was planned to be conducted with patients over 18 years of age but was generally limited to the geriatric age group. This is because hypervolemic patients presented with diagnoses such as renal failure or heart failure and the mean age of patients with these comorbidities was high. Since the US measurements of the study group and the control group were compared, the mean age of the control group patients had to be selected high. The fact that the patients were evaluated in the supine position may have especially affected SCV and IJV diameters. The fact that the emergency medicine residents who participated in our study had only basic US certification and did not receive advanced US training may have caused their manual skills to be insufficient. Therefore, it should be considered that this may have led to the failure to pay attention to compression when evaluating the diameter of superficial veins. The measurements made by two emergency medicine residents were not compared. Our study was a single-center study and had a small sample size. Studies with larger groups should be planned to eliminate these limitations.

## Conclusions

During US evaluations, the maximum and minimum diameters of the IVC, SCV, IJV, and FV were significantly higher in volume-overloaded patients than in normovolemic patients. The IVC-CI was not correlated with the SCV-CI, the IJV-CI, or the FV-CI in volume-overloaded patients; therefore, superficial venous vessels cannot be an alternative to the IVC. The reason for non-correlation may be that superficial veins are subjected to compression when measured with US. In future studies on this subject, the measurement of superficial veins should be performed by clinicians who have received advanced US training and should pay attention to the compression of the veins during measurement.
